# Differential effects of anxiety and autism on social scene scanning in males with fragile X syndrome

**DOI:** 10.1186/s11689-017-9189-6

**Published:** 2017-09-25

**Authors:** Hayley Crawford, Joanna Moss, Chris Oliver, Deborah Riby

**Affiliations:** 1 Centre for Research in Psychology, Behaviour and Achievement, Coventry University, Coventry, CV1 5FB UK; 2 Cerebra Centre for Neurodevelopmental Disorders, School of Psychology, University of Birmingham, Edgbaston, B15 2TT UK; 3 Institute of Cognitive Neuroscience, University College London, 17 Queen Square, London, WC1N 3AR UK; 4 Department of Psychology, Durham University, Durham, DH1 3LE UK

**Keywords:** Eye tracking, Fragile X syndrome, Autism spectrum disorder, Anxiety, Social attention

## Abstract

**Background:**

Existing literature draws links between social attention and socio-behavioural profiles in neurodevelopmental disorders. Fragile X syndrome (FXS) is associated with a known socio-behavioural phenotype of social anxiety and social communication difficulties alongside high social motivation. However, studies investigating social attention in males with FXS are scarce. Using eye tracking, this study investigates social attention and its relationship with both anxiety and autism symptomatology in males with FXS.

**Methods:**

We compared dwell times to the background, body, and face regions of naturalistic social scenes in 11 males with FXS (*M*_age_ = 26.29) and 11 typically developing (TD) children who were matched on gender and receptive language ability (*M*_age_ = 6.28). Using informant-report measures, we then investigated the relationships between social scene scanning and anxiety, and social scene scanning and social communicative impairments.

**Results:**

Males with FXS did not differ to TD children on overall dwell time to the background, body, or face regions of the naturalistic social scenes. Whilst males with FXS displayed developmentally ‘typical’ social attention, increased looking at faces was associated with both heightened anxiety and fewer social communication impairments in this group.

**Conclusions:**

These results offer novel insights into the mechanisms associated with social attention in FXS and provide evidence to suggest that anxiety and autism symptomatology, which are both heightened in FXS, have differential effects on social attention.

## Background

Fragile X syndrome (FXS) is the most common cause of inherited intellectual disability affecting approximately 1 in 2500 males and 1 in 4000–6000 females [1]. FXS is caused by excessive cytosine-guanine-guanine (CGG) repeats on the Fragile X Mental Retardation 1 (FMR1) gene located on the Xq27.3 site. Individuals with the FXS premutation have 45–200 repeats whereas individuals with the full mutation have in excess of 200 repeats. The excessive CGG repeats cause the FMR1 gene to become methylated, resulting in reduced production of the protein FMRP. As FXS is an X-linked disorder, males are more severely affected than females. The phenotype associated with FXS encompasses mild to profound intellectual disability alongside physical, cognitive, and behavioural manifestations [2].

FXS is associated with a socio-behavioural phenotype that includes being motivated to interact with others and demonstrating interest in the social world. However, these features co-occur with heightened anxieties and social communication impairments [2, 3]. The social communication impairment associated with FXS is reflected in the heightened prevalence of autism spectrum disorders (ASD). Although prevalence figures often vary across studies, a recent meta-analysis has indicated that approximately 30% of males with FXS meet criteria for ASD [4]. This is in comparison to 1% of the general population [5]. However, it is increasingly recognised that subtle differences exist between individuals with FXS and those with idiopathic ASD, as those with FXS often display a milder profile of autism symptomatology. A recent review of existing literature highlights several studies indicating less severe social impairments in individuals with FXS and comorbid ASD compared to individuals with idiopathic ASD, particularly on measures of social responsiveness [6].

Anxiety is also commonly reported in FXS with over 80% of males meeting criteria for one anxiety disorder and 60% meeting criteria for multiple anxiety disorders. The most common types of anxiety disorder in FXS are specific phobia, selective mutism, and social phobia. Approximately 60% of males with FXS display clinically significant features of social phobia [7]. Despite social communication impairments and social anxiety, individuals with FXS are reported to show behaviours suggestive of a willingness to interact with others; thus, they appear socially motivated [8–10].

Relevant to the features of FXS described above, existing literature within the field of developmental disorders has drawn links between socio-behavioural characteristics and social attention. Research has primarily identified atypically reduced social attention in ASD (behaviourally associated with social withdrawal) and atypically prolonged social attention in Williams syndrome (WS; behaviourally associated with hyper-sociability) [11–14]. Specifically, this research has demonstrated that people with ASD spend less time than typically developing (TD) individuals viewing people and faces in static pictures of social interaction. Attention to social stimuli in this group has also been linked to social behaviour, with reduced social attention being associated with more severe autism symptomatology and consequently more social communication difficulties [15–17]. Much research has focussed on the association between social behaviour and social attention in ASD. However, little is known about the way in which behavioural characteristics interact with social attention in males with FXS despite the known social profile associated with this group, and the heightened risk of autism.

Studies that have been conducted in FXS have identified atypical social attention, in the form of reduced looking to the eye region of static isolated faces, compared to TD individuals [18–20] and individuals with ASD [20, 21]. However, every one of these studies used isolated face images displaying different emotional expressions. Whilst this offers rich information regarding looking patterns to facial features in FXS, it is known from the literature on both typical development and ASD that such stimuli lack ecological validity as there is no ‘competition’ between social and non-social attention capture (e.g. see discussions by [16]). One study that has investigated social attention to more naturalistic social scenes reported that a largely female sample of people with FXS spent a ‘typical’ amount of time looking at social information, but that they also looked away quicker than TD participants, indicating active social avoidance [22]. The issue that 12 out of the 14 FXS participants in that study were female is important due to the striking differences in the severity and prevalence of the FXS phenotype between males and females. Therefore, it is problematic to generalise findings from studies using largely female samples to males with FXS who are often more severely affected.

There is a need to utilise ecologically valid social scene stimuli to understand the social attention of males with FXS. Furthermore, given the socio-behavioural profile of the disorder, preliminary insight into the role of anxiety and autistic features is important to understand the potential mechanisms underlying social attention in this group. In typical development, it is known that socially anxious individuals fixate longer on the eye region of faces than those without social anxiety [23]. Anxiety has previously been related to social attention in people with WS, but in a different way, with high levels of anxiety being associated with reduced fixation on faces and eye regions of threatening facial expressions [24]. In FXS, some studies have reported that reduced fixation to the eye region of isolated emotionally expressive faces is not associated with social anxiety [20] or autism symptomatology [19, 21], whereas other studies have reported a positive correlation between self-reported social anxiety and time spent looking at the eye region of faces [25]. Studying FXS, a genetic syndrome with heightened risk of autism and anxiety, offers novel insight into the association between these behavioural characteristics and social attention, which may inform understanding of other neurodevelopmental disorders associated with a similar socio-behavioural profile, e.g. ASD and Cornelia de Lange syndrome [26].

Whilst existing eye-tracking studies in FXS have offered rich information regarding the extent of eye gaze aversion, the current study makes a significant contribution to investigating the influence of anxiety and autism symptomatology on social attention in males with FXS using naturalistic social scenes that reflect the complexities of our social world. This study aims to (1) compare and contrast social attention in males with FXS to TD children matched on gender and receptive language ability, (2) investigate the relationship between social attention and anxiety in males with FXS, and (3) investigate the relationship between social communication impairment and social attention in males with FXS.

## Methods

### Participants

Participants were 11 males with FXS aged between 14 and 43 years (*M*_age_ = 26.29; 9.06). All participants had a confirmed diagnosis from a professional (paediatrician, general practitioner, or clinical geneticist). Participants with FXS were recruited through the Cerebra Centre for Neurodevelopmental Disorders participant database at the University of Birmingham.

Participants with FXS were group-matched to 11 male TD children on receptive language ability (*t* (20) = −1.208, *p* = .242) using the raw scores from the British Picture Vocabulary Scale (BPVS; [27]). As previous literature indicates that receptive language is commensurate with nonverbal mental age in adolescents with FXS [28], receptive language was used as a proxy indicator of general intellectual ability. TD children were recruited through the Infant and Child Laboratory participant database, also at the University of Birmingham. None of the TD children scored above 15 on the Social Communication Questionnaire (SCQ; [29]), the score suggested by the authors to be indicative of ASD. All of the TD children scored within the normal range on the Spence Child Anxiety Scale—Parent version (SCAS-P; [30]), defined as the mean + 1 standard deviation, using the national normal data from TD boys aged 6–11 years [31]. The same criterion was used to rule out anxiety in children under the age of 6 years in the current study. Table 1 presents the final participant characteristics.

**Table 1:** Participant characteristics and alpha level for comparison between FXS and TD participants

	FXS (*n =* 11)	TD (*n =*11)	*t*	df	*p*
Chronological age (years)					
Mean (SD)	26.29 (9.06)	6.28 (1.31)	−7.256	20	<.001
Range	14.12–43.01	4.60–8.94			
Receptive language ability (raw score)					
Mean (SD)	87.00 (27.21)	74.18 (22.32)	−1.208	20	.241
Range	87–135	47–114			
Gender (% male)	100	100			

Comparison between participants on chronological age, receptive language ability as measured by the British Picture Vocabulary Scale, and gender

All participants had normal or corrected to normal vision. All participants aged 16 years and over provided informed written consent, and parents of children aged under 16 provided written consent before taking part in the study, in line with the ethical approval granted from the Science, Technology, Engineering and Mathematics Ethical Review Committee at the University of Birmingham.

### Stimuli and apparatus

The stimuli used were identical to those used by Riby and Hancock [11]. Stimuli consisted of 20 colour photographs of naturalistic social scenes including human actors engaged in natural activities. Example scenes included a bride and groom on their wedding day, a woman on the phone, a group of friends talking to one another, and a teacher in a classroom. Actors in the photographs were not directing their attention towards the camera and displayed natural facial expressions. Specifically, the emotional valence of the actors in the social scenes was mostly neutral, interspersed with a few images where actors were displaying a happy facial expression. The scenery was naturalistic for the activities that actors were engaged in, e.g. classroom, restaurant. Participants also saw five filler photographs of landscapes with no actor, which were interspersed throughout the eye-tracking task so as to avoid a uniform pattern of solely social scenes being displayed. As filler trials contained no social stimuli, eye movements during these trials were not analysed. Stimuli were 640 × 480 pixels.

Stimuli were presented on a 24-in. widescreen LED monitor at a screen resolution of 1680 × 1050. Participants’ eye movements were recorded using an EyeLink 1000 Tower Mount system, which runs with a spatial accuracy of .5–1 visual angle (°), a spatial resolution of .01°, and a temporal resolution of 500 Hz. The right eye of each participant was tracked. The eye-tracking camera was linked to a host PC separate to the one displaying the stimuli. EyeLink software (SR research, Ontario, Canada) was used to control the camera and collect data.

### Measures

The participants' primary caregivers completed the SCQ [29] and the SCAS-P [30] to measure social communication impairments and anxiety, respectively, and for the purposes of investigating associations between these behavioural characteristics and social attention in the present study. The SCAS-P assesses the following six domains of anxiety: physical injury fears, obsessive-compulsive disorder, separation anxiety, social phobia, panic/agoraphobia, and generalised anxiety, and has been shown to differentiate those with and without an anxiety disorder. Internal consistencies of the total scale and subscales range from .83 to .92 in an anxiety-disordered group and .81 to .90 in typical controls. The SCAS-P total score correlates significantly with the Child Behavior Checklist [32] internalising subscale, indicating convergent validity [31]. Caregivers completed these measures either whilst their child was participating in the study or at home, returning it to the researchers on completion. All participants lived at home with the caregiver completing the questionnaire measures. The Autism Diagnostic Observation Schedule (ADOS; [33]) was administered to all participants with FXS for diagnostic purposes (module 2: *n* = 2; module 3: *n* = 5; module 4: *n* = 4). The BPVS [27] was administered to all participants to assess receptive language ability.

### Procedure

Participants were tested individually at the University of Birmingham in a dimly lit room with windows blacked out to avoid luminance changes. Participants were seated approximately .6 m from the screen with their chin resting on a chinrest and their forehead against a headrest. The chinrest and desk height were adjusted so that eye gaze was central to the display screen. A 5-point calibration was performed prior to the experiment during which participants followed the location of an animated blue dolphin positioned at the edges of the display area. The calibration procedure was repeated until successful, and all participants included in the analysis achieved a full 5-point calibration. Following calibration, the participants were told that they would view a series of pictures and that they could look wherever they wished whilst these were displayed. Each image was then presented for 5 s. Between each trial, a fixation cross appeared at the centre of the screen for 1 s.

### Data analysis

Areas of interest (AOI) were designated to the face, body, and background using the Data Viewer programme (SR Research). Face and body AOI were created using the FreeHand Interest Area Shape to select the outline of each actor’s face and body. The background AOI was created using the Rectangular Interest Area Shape, to cover the entire image, and then subtracting fixation data from the face and body AOI prior to analysis. Data are presented as the total time, in milliseconds, that fixations were within each AOI. A trial was deemed invalid, and therefore excluded, if a participant did not look at the picture presented for any of the trial time. If any participant produced more than 40% invalid trials, their data were excluded from analyses. In the current study, one participant produced one invalid trial only. Therefore, no participants were excluded due to insufficient data. All data were subjected to the Shapiro-Wilk test for normality. Where data were not normally distributed, non-parametric tests were used for statistical analyses. For the between-group comparisons, where results from non-parametric tests did not differ from results from the equivalent parametric tests, the results from the parametric tests are reported. For within-group correlations, Spearman’s correlations are used where data are not normally distributed and Pearson’s correlations are used where data are normally distributed. The alpha level for significance was .05.

## Results

**Fig. 1:**
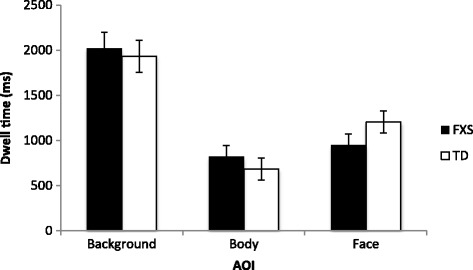
Dwell time on AOIs; dwell time in milliseconds on background, body, and face AOI for the FXS and TD participant groups, when overall engagement with the stimuli did not differ across groups

There was no difference in the overall amount of time participants spent viewing stimuli, indicating comparable task engagement across the groups (FXS mean per image: 4202.46 ms; TD mean per image: 4237.88 ms; *t* (20) = .148, *p* = .884). The remaining analyses concern dwell time in milliseconds for each AOI (see Fig. 1).

A 3 (AOI: background, body, face) × 2 (group: FXS, TD) ANOVA was conducted, which revealed a significant main effect of AOI (*F* (2, 40) = 38.153, *p* < .001, *n* ^2^ = .656) but no significant main effect of group (*F* (1, 20) = .009, *p* = .923, *n* ^2^ < .001), and no significant interaction (*F* (2, 40) = 1.066, *p* = .354, *n* ^2^ = .051). Bonferroni post hoc tests indicated that the main effect of AOI was driven by longer dwell time on the background than the body and the face regions of the actors in the scenes (both *p* < .001). Dwell times on the face and body region of actors were statistically comparable (*p* = .081). However, Wilcoxon signed rank tests, which were conducted because body AOI data were not normally distributed, revealed longer dwell time on the face compared to the body region of actors (*Z* = −2.029, *p* = .042).

**Fig. 2:**
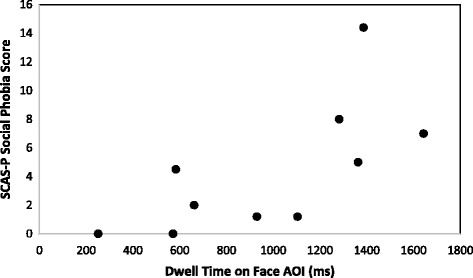
Relationship between face AOI and social anxiety; a scatterplot depicting the relationship between dwell time on the face AOI in milliseconds, and the SCAS-P social phobia score for participants with FXS. The analyses indicate a significant positive correlation (*r*_p_ (8) = .687, *p* = .028)

**Fig. 3:**
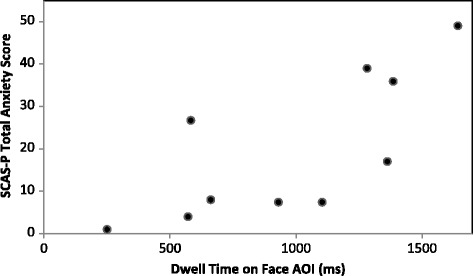
Relationship between face AOI and anxiety; a scatterplot depicting the relationship between dwell time on the face AOI in milliseconds, and the SCAS-P total score for participants with FXS. The analyses indicate a significant positive correlation (*r*_p_ (8) = .742, *p* = .014)

**Fig. 4:**
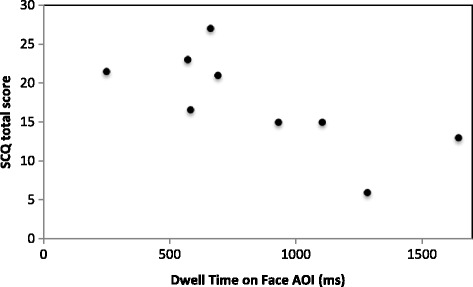
Relationship between face AOI and autism symptomatology; a scatterplot depicting the relationship between dwell time on the face AOI in milliseconds and the SCQ total score for participants with FXS. The analyses indicate a significant negative correlation (*r*_p_ (7) = −.720, *p* = .029)

Correlations were conducted to assess the association between dwell time on each AOI and social communication difficulties, as measured by the SCQ, and social phobia and total anxiety scores, as measured by the SCAS-P for each participant group. Table 2 shows the descriptive statistics for these measures by group. A significant negative correlation between SCQ score and dwell time to the background was revealed for the TD group (*r*_s_ (7) = −.792, *p* = .011), indicating that those individuals with fewer social communication difficulties spent more time looking at the background. No other significant correlations were revealed for the TD participant group (all *p* > .05; Table 3). For participants with FXS, moderate-strong positive correlations were revealed between dwell time on the face AOI and social phobia (*r*_p_ (8) = .687, *p* = .028; Fig. 2), and between dwell time on the face AOI and total anxiety score (*r*_p_ (8) = .742, *p* = .014; Fig. 3). A significant negative correlation was revealed between dwell time on the face AOI and SCQ score (*r*_p_ (7) = −.720, *p* = .029; Fig. 4). This did not remain significant after controlling for receptive language ability (*r*_p_ (5) = −.704, *p* = .077). Taken together, this indicates that those FXS participants with higher anxiety scores, and fewer social communication difficulties, exhibited longer dwell times on faces.

**Table 2:** Descriptive statistics and alpha level for the ADOS, SCQ, and SCAS-P measures

Measure	FXS	TD	*t*	df	*p*
ADOS					
Mean raw total score (SD)	8.64 (5.12)	NA			
Range	2–22				
% meeting cut-off for ASD	72.73				
% meeting cut-off for autism	18.18				
Social Communication Questionnaire^a^					
Mean raw total score (SD)	17.57 (6.27)	2.89 (2.37)	−6.569	16	<.001
Range	6–27	0–6			
% meeting cut-off for ASD	77.7	0			
% meeting cut-off for autism	22.22	0			
Spence Child Anxiety Scale^b^					
Mean raw Social Phobia score (SD)^c^	4.33 (4.53)	2.63 (2.26)	−.967	16	.348
Range	0–14.4	0–6			
Mean raw total score (SD)^d^	19.54 (16.95)	9.38 (5.32)	−1.625	16	.124
Range	1–49	1–20			

^a^SCQ was not completed for two TD participants and two FXS participants

^b^SCAS-P was not completed for three TD participants and one FXS participant

^c^The maximum Social Phobia score on the SCAS-P is 18. Normative data obtained from Nauta et al. [31] indicate a mean score of 7.3 for anxiety-disordered and a mean score of 4.3 for typically developing boys aged 6–11 years

^d^The maximum total score on the SCAS-P is 114. Normative data obtained from Nauta et al. [31] indicate a mean total score of 31.4 for anxiety-disordered and 16.0 for typically developing boys aged 6–11 years

**Table 3:** Correlations between behavioural characteristics and social attention, and between participant characteristics and social attention

	Fragile X syndrome			Typically developing		
	Face *r*_p_ (*p*)	Body *r*_p_ (*p*)	Background *r*_p_ (*p*)	Face *r*_p_ (*p*)	Body *r*_s_ (*p*)	Background *r*_s_ (*p*)
Social phobia	.687 (.028)	−.311 (.981)	−.161 (.657)	−.059 (.890)	−.024 (.955)	−.539 (.168)
Total anxiety score	.742 (.014)	−.153 (.673)	−.250 (.486)	−.265 (.525)	.120 (.776)	−.663 (.073)
Total SCQ score	−.720 (.029)	.077 (.845)	.099 (.800)	−.660 (.053)	.017 (.965)	−.792 (.011)
Chronological age	.593 (.055)	−.105 (.758)	.165 (.627)	.166 (.627)	−.082 (.811)	.191 (.574)
Receptive language	.383 (.246)	.073 (.831)	.422 (.196)	−.178 (.601)	.178 (.601)	.483 (.132)

Correlation matrix for correlations between dwell time on face, body, and background AOI with (1) social phobia, as measured by the SCAS-P, (2) total anxiety score on the SCAS-P, (3) social communication impairment, as measured by the SCQ, (4) chronological age, and (5) receptive language raw score, as measured by the BPVS

As participant groups were not matched on chronological age, correlations were conducted to assess the relationship between chronological age and dwell time, especially due to the large age range of the FXS group. These revealed no significant association between chronological age and dwell time on any AOI for either participant group (all *p* > .05). Although participant groups were matched on receptive language ability, correlations were conducted to assess the relationship between receptive language and dwell time in the event that our group-matching comparison was underpowered. These revealed no significant association between receptive language and dwell time on any AOI for either participant group (all *p* > .05).

## Discussion

In the present study, we examined and compared visual attention to naturalistic social scenes in males with FXS versus TD individuals. In addition, we investigated the relationship between social attention, anxiety, and social communication difficulties. The results demonstrated statistically comparable dwell time on background, body, and face regions of the social scenes across the two participant groups. The results also demonstrated an association between increased looking at faces with increased anxiety and fewer social communication difficulties in individuals with FXS. Together, these results suggest that whilst social attention to naturalistic social scenes may be developmentally ‘typical’ in males with FXS, anxiety and autism symptomatology are differentially related to social attention in this population.

Existing studies that have indicated atypical social attention in males with FXS have focussed on attention to the eye region of static faces. However, the current study revealed that social attention to naturalistic social scenes appears developmentally ‘typical’ in males with FXS. A number of important advances have indicated reduced social attention in individuals with ASD, which is associated with social withdrawal [11–14]. The milder profile of social communication difficulties, and subtle but important differences in the social impairment reported in individuals with FXS [2, 3, 6], may account for the results presented here, documenting that these individuals do not show reduced social attention in the same way as those with ASD. Existing literature suggests that individuals with FXS demonstrate less severe impairments in social responsiveness compared to individuals with ASD, even when matched on overall autism severity [6, 34]. These different profiles go some way to explaining why reduced social attention may be expected in individuals with ASD but not in those with FXS.

Although there were no significant differences between the FXS and TD groups in relation to overall looking time, increased looking to faces was correlated with fewer social communication difficulties in individuals with FXS. This is a finding that is often reported in the ASD literature [15–17], and one that suggests autism symptomatology may play a role in the viewing of naturalistic social scenes. Interestingly, in our previous work directly comparing individuals with FXS and ASD, we reported that atypical eye gaze in FXS was not a product of autistic symptomatology [21]. Together, these results suggest that social attention to naturalistic scenes appears developmentally typical but may be influenced by autism symptomatology, whereas eye gaze aversion is a FXS-specific impairment that is unlikely to be a product of autism symptomatology in the same way.

The current study reported a relationship between heightened looking at faces and anxiety. A potential mechanism underlying this explanation is that individuals experiencing anxiety, and social anxiety in particular, may view faces as a more threatening aspect of a social scene. Therefore, heightened looking to threatening stimuli may reflect hyper-vigilance for threatening stimuli, supporting previous literature indicating that socially anxious TD individuals fixate longer on the eye region of faces than those without social anxiety [23]. This potential explanation is supported by our previous eye-tracking study, which revealed a positive relationship between social dwell time on videos of actors approaching the viewer, and anxiety, in males with FXS [35]. The results of the current study are also interesting in light of existing behavioural observation research that highlighted a pattern of results in which more eye contact was associated with increased cortisol reactivity, a physiological indicator of stress, in individuals with FXS [36]. It is important to note that although the mean anxiety scores for participants with FXS did not differ from normative data from TD children, within-syndrome variability was large. Participants with FXS were therefore more likely to achieve scores on the SCAS-P indicative of more severe anxiety than children with an anxiety disorder (see [31] for normative data).

The differential relationships reported here, between social attention and both anxiety and autism symptomatology, are particularly interesting when existing literature on WS is considered. Less time spent looking at the eye region of faces has been related to higher levels of autism symptomatology in individuals with WS [37], a similar relationship to that reported in the current study where less looking at faces was associated with higher levels of autism symptomatology. Additionally, increased levels of generalised anxiety have been associated with reduced fixation on faces and eyes for individuals with WS [24], which is the opposite pattern of results to that reported in the current FXS sample where increased levels of anxiety were associated with increased dwell time on faces. One possible explanation for these cross-syndrome differences in the relationship between social attention and anxiety may be related to the different profiles of anxiety associated with these two genetic syndromes. Although both FXS and WS are associated with high levels of specific phobia, FXS is also typically associated with social anxiety [7] whilst WS is associated with generalised anxiety disorder [38]. Such cross-syndrome insights allow us to advance our understanding of syndrome-specific mechanisms that might underlie social attention patterns.

It is essential to apply caution when interpreting the results of the present study due to the small sample sizes. However, moderate to strong correlations between social attention, anxiety, and social communication impairments were revealed even with these small samples, highlighting the potential utility of further investigations in this area. The scatterplots (Figs. 2 and 3) indicate further that the significant correlations are unlikely to be driven by outliers. Whilst the between-group comparisons may have been statistically underpowered, the alpha levels are well above the significance cut-off (group × AOI interaction: *p* = .354; between-group comparisons: *p* = .923). Therefore, it seems unlikely that these results would differ with additional participants.

In addition, the wide age range of the FXS group should be considered when interpreting the results due to the possibility of age-related differences in social attention and behavioural characteristics. Group matching on chronological versus mental age is a common issue in intellectual disability research, and we, therefore, suggest our results indicate *developmentally* ‘typical’ social attention in FXS. The extent to which social attention in the FXS group would compare to individuals of the same chronological age is beyond the scope of this study. However, correlations to investigate the relationship between chronological age and social attention were not significant. Existing literature has reported interesting differences in social attention as a function of chronological age, with children aged 3 months looking more at eyes, and older children aged 30 months looking more flexibly at mouths (especially when talking) and hands (especially when picking up an object) [39]. The development of social attention across childhood and adolescence has focussed on specific skills such as facial expression recognition, which seems to improve with age [40, 41]. Less is known about the effect of age and social experience on social attention in a passive viewing task.

It is important to note that the sample size and age range in the current study is similar to that of other eye-tracking studies investigating social attention in FXS [18–20, 42]. However, further research in this area is required to clarify the nature of social attention to naturalistic social stimuli in males with FXS, and to disentangle the effects of developmental level and other behavioural characteristics, such as social communication impairments and anxiety, on social attention.

Furthermore, although IQ measures were not administered for the present study due to methodological impracticality of administering multiple different IQ tests to account for the wide range of ages and abilities of participants, the two participant groups were matched on receptive language. Receptive language has been reported to be commensurate with nonverbal mental age in adolescents with FXS [28]. It is possible that the statistical test to confirm that groups were matched was underpowered. To that end, receptive language ability was taken into account with our statistical tests, and correlations between receptive language and social attention were not significant. Finally, although genetic reports were not available for the current study, future research could investigate the relationship between genetic factors and social attention. Interestingly, our previous work has demonstrated a relationship between genetic variation and visual scanning of emotional faces [43]. Overall looking time indicated good levels of task engagement by both groups, highlighting the opportunities afforded by using eye tracking to investigate the mechanisms subserving clinically relevant behaviours in males with FXS.

## Conclusions

The present study documents differential effects of anxiety and autism on social attention in males with FXS. To our knowledge, this is the first study to investigate visual attention to naturalistic social scenes in a sample of males with FXS. This offers insights into the potential mechanisms subserving social attention in this population and how this might differ to other genetically defined neurodevelopmental disorders. The research paves the way for future investigations of the relationship between clinically relevant, socio-behavioural phenotypes, and social attention, in theories of social attention in neurodevelopmental disorders.

## Abbreviations

ADOS: Autism Diagnostic Observation Schedule
ANOVA: Analysis of variance
AOI: Area of interest
ASD: Autism spectrum disorder
BPVS: British Picture Vocabulary Scale
CGG: Cytosine-guanine-guanine
FMR1: Fragile X Mental Retardation 1 gene
FMRP: Fragile X Mental Retardation Protein
FXS: Fragile X syndrome
SCAS-P: Spence Child Anxiety Questionnaire—Parent Version
SCQ: Social Communication Questionnaire
TD: Typically developing
WS: Williams syndrome
